# Corticostriatal functional connectivity of bothersome tinnitus in single-sided deafness

**DOI:** 10.1038/s41598-019-56127-1

**Published:** 2019-12-20

**Authors:** Jennifer Henderson-Sabes, Yingying Shang, Philip L. Perez, Jolie L. Chang, Seth E. Pross, Anne M. Findlay, Danielle Mizuiri, Leighton B. Hinkley, Srikantan S. Nagarajan, Steven W. Cheung

**Affiliations:** 1 Department of Otolaryngology–Head and Neck Surgery, University of California, San Francisco, USA; 2 Department of Otorhinolaryngology, Peking Union Medical College Hospital, Beijing, China; 3 Department of Radiology and Biomedical Imaging, University of California, San Francisco, USA

**Keywords:** Diagnostic markers, Cortex

## Abstract

Subjective tinnitus is an auditory phantom perceptual disorder without an objective biomarker. Bothersome tinnitus in single-sided deafness (SSD) is particularly challenging to treat because the deaf ear can no longer be stimulated by acoustic means. We contrasted an SSD cohort with bothersome tinnitus (TIN; N = 15) against an SSD cohort with no or non-bothersome tinnitus (NO TIN; N = 15) using resting-state functional magnetic resonance imaging (fMRI). All study participants had normal hearing in one ear and severe or profound hearing loss in the other. We evaluated corticostriatal functional connectivity differences by placing seeds in the caudate nucleus and Heschl’s Gyrus (HG) of both hemispheres. The TIN cohort showed increased functional connectivity between the left caudate and left HG, and left and right HG and the left caudate. Within the TIN cohort, functional connectivity between the right caudate and cuneus was correlated with the Tinnitus Functional Index (TFI) relaxation subscale. And, functional connectivity between the right caudate and superior lateral occipital cortex, and the right caudate and anterior supramarginal gyrus were correlated with the TFI control subscale. These findings support a striatal gating model of tinnitus and suggest tinnitus biomarkers to monitor treatment response and to target specific brain areas for innovative neuromodulation therapies.

## Introduction

Tinnitus is a common auditory phantom perceptual disorder where conventional audiometric hearing loss profiles alone cannot help clinicians to distinguish between patients who merely experience tinnitus from those who are troubled by tinnitus^1,2^. The search for physiological substrates that account for tinnitus persistence and tinnitus severity has led investigators to evaluate the central nervous system (CNS) using a variety of techniques. Some documented CNS changes are synchronous hyperactivity^3–5^, tonotopic map cortical plasticity^6–8^, thalamocortical dysrhythmia^9,10^, and gamma band oscillations^11–13^.

Human physiological studies^14,15^, case reports^16,17^, and an early clinical trial^18^ focused on the caudate nucleus of the basal ganglia support a striatal gating model^15^ of tinnitus awareness. This model delineates modulators of tinnitus persistence and tinnitus severity, where corticostriatal connections between the striatum and auditory cortex act to gate auditory phantom representations to reach perceptual awareness and connections between the striatum and limbic structures act to modulate auditory phantom distress.

A striatal gating model of phantom percept awareness is complementary to other CNS hypotheses, including those that posit tinnitus is primarily an expectation mismatch within the auditory system^19,20^ or is driven by abnormal auditory-limbic interactions^21–23^. Neuroimaging studies in support of abnormal striatal connectivity as a potential biomarker of chronic tinnitus^24,25^ have been reported in cohorts with inhomogeneous hearing loss profiles. Those studies used post-hoc statistical techniques to address the possible confound of hearing loss levels on neural correlates of tinnitus. However, there is not yet a neuroimaging investigation that incorporates a specific hearing loss pattern in a cohort contrast study design that could isolate differential network connectivity findings to chronic tinnitus.

Patients with bothersome tinnitus in single-sided deafness (SSD) or unilateral severe to profound hearing loss and normal or nearly normal thresholds in the only hearing ear represent an exceptional opportunity to study tinnitus not confounded by hearing loss variations. Bothersome tinnitus in adult acquired SSD is expected to be localized to the deaf ear^2,26^, although this is not necessarily the case in congenital SSD^27^. Tinnitus localized to the deaf ear eliminates the treatment option of masking sound delivery to the defective sensory end organ, as it is unresponsive to acoustic stimulation. Moreover, acoustic therapies (amplification, masking, customized acoustic stimuli) directed to the better hearing ear are of minimal to no benefit^28–30^. Behavioral therapies (Tinnitus Retraining and Cognitive Behavioral) may be beneficial in modulating tinnitus distress, but without effective sound therapy to the deaf ear, have little to no effect on tinnitus loudness^31,32^. Neuromodulation of the auditory periphery by cochlear implantation of the deaf ear, an alternative method of auditory system stimulation, often reduces tinnitus severity similar to acoustic therapies in an ear with hearing loss^33–36^. However, this intervention requires surgical implantation of hardware to the skull and complicates future head magnetic resonance imaging (MRI) examinations.

The goal of this study was to identify candidate biomarkers to monitor tinnitus treatment response and targets for brain-based neuromodulation approaches. We evaluated basal ganglia and cortical connectivity patterns by contrasting an SSD cohort with bothersome tinnitus (TIN) against an SSD cohort no or non-bothersome tinnitus (NO TIN). We used resting-state functional magnetic resonance imaging (fMRI) to define whole-brain connectivity patterns of the caudate nucleus and auditory cortex. We report on connectivity differences between TIN and NO TIN cohorts, and voxelwise connectivity strength correlations with subscale scores of the validated Tinnitus Functional Index (TFI)^37^ within the TIN cohort.

## Results

### Demographics and audiometrics

**Figure 1:**
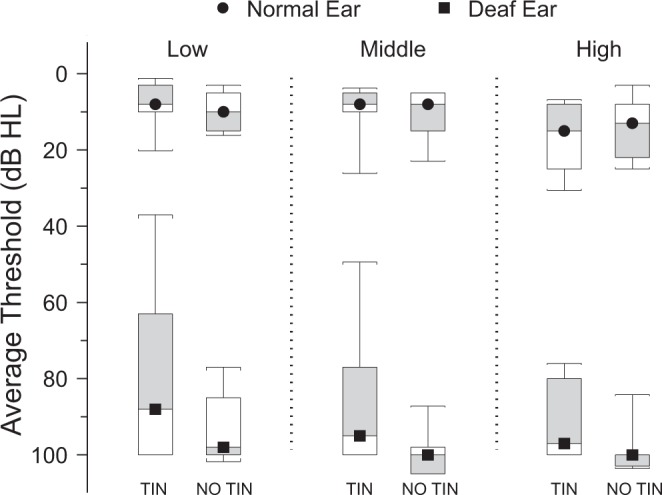
Audiometric threshold Tukey boxplots of low frequency (average of 250 and 500 Hz), middle frequency (average of 1000 and 2000 Hz) and high frequency (average of 4000, 6000 and 8000 Hz) bands of normal (circle) and deaf (square) ears for both cohorts show no significant differences across all bands. TIN – bothersome tinnitus. NO TIN - no or non-bothersome tinnitus (NO TIN).

Data on TFI score, age, sex, deafness duration, diagnosis of vestibular schwannoma, and deaf ear laterality for SSD TIN and SSD NO TIN cohorts are shown in Table 1. Descriptive statistics, using mean (standard error) and ratio conventions were computed for each cohort. The two cohorts differed only in TFI score (t-test, p < 0.001), an expected result of the study design; all other comparisons were not significantly different. Pure tone audiometric thresholds for low, middle, and high frequency bands of the normal and deaf ears were not significantly different for the two cohorts (Fig. 1). The mean (standard error) of the three frequency bands were as follows: Low (normal ear): TIN = 9.1 (2.3), NO TIN = 10.3 (1.5); Low (deaf ear): TIN = 78.5 (3.3), NO TIN = 89.5 (5.2). Middle (normal ear): TIN = 11.0 (2.2), NO TIN = 11.0 (1.8); Middle (deaf ear): TIN = 86.3 (5.3), NO TIN = 98.6 (2.3). High (normal ear): TIN = 15.9 (2.5), NO TIN = 15.8 (3.1); High (deaf ear): TIN = 91.8 (3.0), NO TIN = 97.7 (2.3). When thresholds exceeded the limits of the audiometer, the limit of the equipment (100–110 dB) was used for analysis.

**Table 1:** Single-Sided Deafness Cohorts.

Cohort	Total TFI score	Age (Years)	Sex	Deafness (Years)	VS Tumor	Deaf Ear
SSD TIN	13	55	F	7	No	Left
	13	52	F	1.5	Yes	Left
	14	56	M	2	No	Right
	15	62	F	2	No	Right
	18	31	M	11	No	Right
	22	62	F	14	No	Right
	24	44	M	2.6	Yes	Right
	30	47	M	5	No	Left
	39	57	F	2	No	Left
	56	44	M	9	Yes	Right
	67	54	M	45	No	Left
	72	44	F	2	No	Left
	73	46	M	1.2	Yes	Left
	84	51	M	19	No	Right
	85	61	M	10	Yes	Right
	41.7 (7.2)^†^	51.1 (2.2)	M:F = 9:6	8.9(2.9)	Y:N = 5:10	L:R = 7:8
SSD NO TIN	0	44	M	2	No	Right
	0	65	F	12	Yes	Right
	0	24	M	1	No	Right
	0	77	M	8	No	Right
	0	25	F	25	No	Left
	0	61	M	17	No	Right
	0	57	F	8	No	Left
	0	36	M	25	No	Right
	0	39	M	1.3	Yes	Right
	1	24	M	18	No	Right
	3	48	F	2	Yes	Left
	4	59	M	10	Yes	Right
	5	27	F	3	No	Left
	12	59	M	3	No	Left
	12	63	F	2.5	No	Right
	2.5(1.1)	47.2(4.5)	M:F = 9:6	9.2(2.2)	Y:N = 4:11	L:R = 5:10

SSD – Single-sided deafness. TIN – Bothersome tinnitus. NO TIN – No or non-bothersome tinnitus. VS – Vestibular schwannoma. Descriptive statistics convention is mean (standard error). ^†^SSD TIN vs SSD NO TIN comparison is statistically significant (t-test, p < 0.001). All participants were provided with TFI, those with a score of 0 rated awareness of tinnitus at 0%.

### Bothersome vs non-bothersome tinnitus

**Figure 2:**
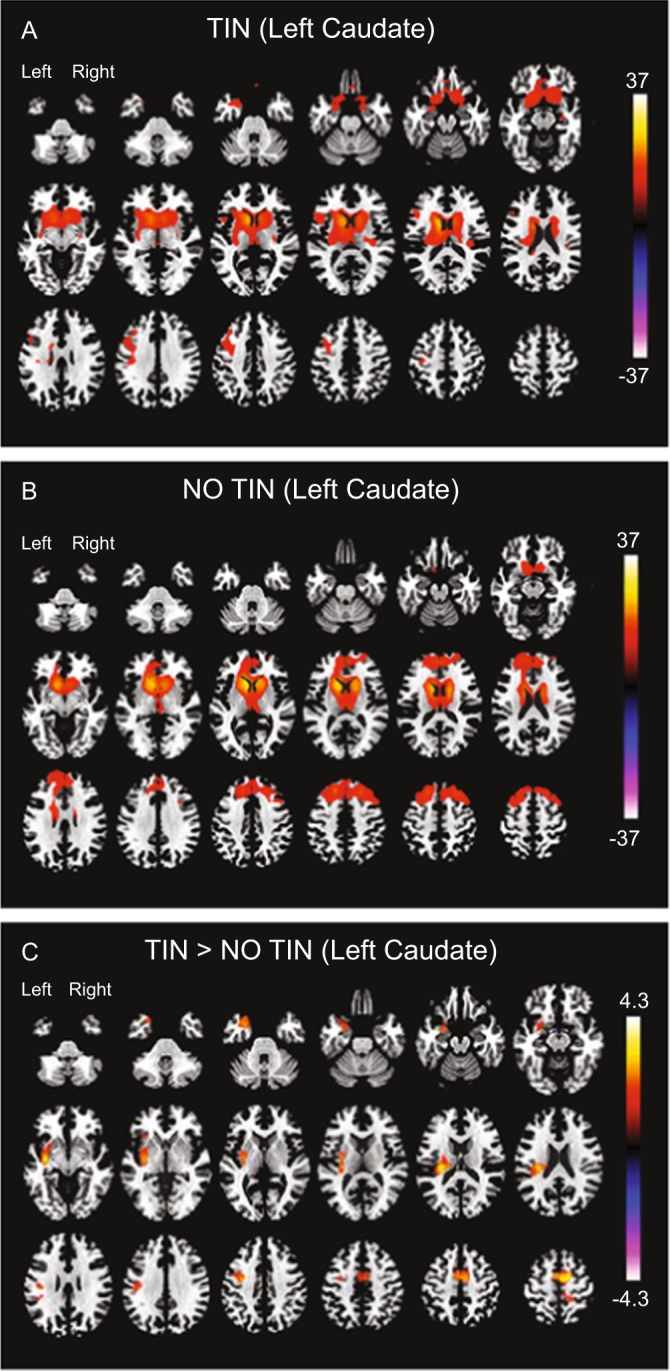
Functional connectivity of the left caudate in both cohorts. (**A**) TIN within-group connectivity. (**B**) NO TIN within-group connectivity. (**C**) TIN > NO TIN connectivity differences. The TIN cohort exhibits increased functional connectivity from the left caudate to left HG (corticostriatal) and right supplementary motor area. The color bar represents the t-statistic of differences in functional connectivity within (**A**,**B**) or between (**C**) cohorts. Positive values (red colors) indicate increased connectivity and negative values (blue colors) indicate decreased connectivity. HG – Heschl’s gyrus.

Resting-state functional connectivity maps were reconstructed for TIN and NO TIN cohorts for the four seed regions (Heschl’s gyrus (HG), and caudate, bilaterally). Functional connectivity seeded from the left caudate was significant with the right caudate, and with multiple regions of pre-frontal cortex (PFC) in both TIN and NO TIN cohorts (Fig. 2A,B). The TIN cohort exhibited increased functional connectivity between the left caudate and a region of the left HG/insula, and between the left caudate and the right supplementary motor area (Fig. 2C). There were no differences between the two cohorts in functional connectivity for the other three seed regions (right caudate, right HG, and left HG). Table 2 lists the centroid coordinates of regions with increased functional connectivity referenced to ROI seeds, in compliance with reporting standards for neuroimaging studies.

**Table 2:** Connectivity Strength Differences between SSD TIN and NO TIN Cohorts.

Region of Interest	MNI Coordinates [X Y Z]	Anatomical Label
Left Caudate (increased)	[10 −6 58]	Right supplementary motor area
	[−30 −24 18]	Left Heschl’s Gyrus/PO

SSD – Single-sided deafness. TIN – Bothersome tinnitus. NO TIN – No or non-bothersome tinnitus. MNI - Montreal Neurological Institute. PO – parietal operculum. All p < 0.005 (false discovery rate cluster-mass corrected).

### TFI subscale correlation with functional connectivity

**Figure 3:**
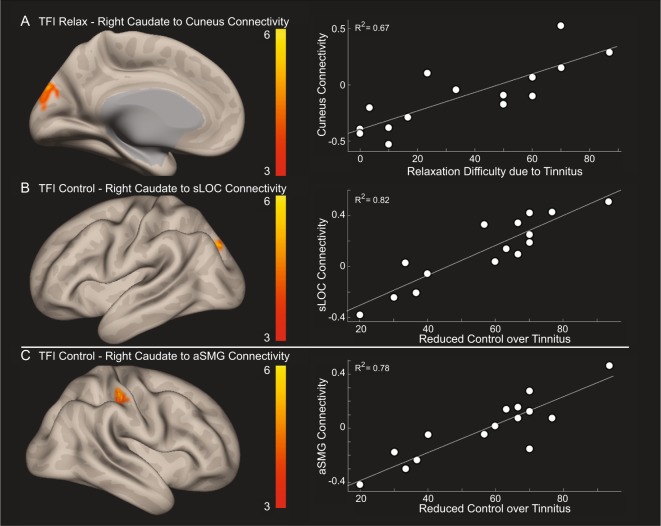
Within the TIN cohort, connectivity strength (regression t-score) between the caudate nucleus and other nonauditory structures is correlated with a specific TFI subscale score. (**A**) Right caudate nucleus to cuneus connectivity is significantly correlated with relaxation difficulty due to tinnitus. (**B**,**C**) Right caudate nucleus to superior lateral occipital cortex (sLOC) and to anterior supramarginal gyrus (aSMG) is correlated with sense of reduced control over tinnitus. Yellow indicates significantly increased connectivity. TFI – Tinnitus Functional Index.

Within the TIN cohort, voxelwise correlations with TFI subscales showed increased connectivity between the right caudate and cuneus for the relaxation subscale, where increased connectivity was correlated with higher interference with the ability to relax (Fig. 3). Voxelwise correlations analysis also showed increased connectivity between the right caudate and superior lateral occipital cortex and the right caudate and anterior supramarginal gyrus for the control subscale, where increased connectivity was correlated with the sense of reduced control over the tinnitus percept (Fig. 3). No other TFI subscale scores showed statistically significant correlations with right caudate connectivity. Table 3 lists centroid coordinates of brain areas with increased connectivity to the right caudate for the relaxation and control TFI subscales.

**Table 3:** Connectivity Strength Correlations with TFI Subscales in SSD TIN Cohort.

Region of Interest	TFI Subscale	MNI Coordinates [X Y Z]	Anatomical Label
Right Caudate	Relax	[−6 −88 34]	Occipital pole/Cuneus
	Control	[−30 −86 36]	Superior lateral occipital cortex
	Control	[48 −32 4]	Anterior supramarginal gyrus

SSD – Single-sided deafness. TIN – Bothersome tinnitus. NO TIN – No or non-bothersome tinnitus. MNI - Montreal Neurological Institute. All p < 0.05 (false discovery rate cluster-mass corrected).

## Discussion

The key finding of this study is increased connectivity between the caudate nucleus and auditory cortex in the SSD cohort with chronic, bothersome tinnitus. All patients relied on monaural hearing with normal audiometric thresholds, thereby removing hearing loss variation as a possible confound and isolating abnormal striatal functional connectivity to chronic tinnitus. This independent replication of the critical finding in our prior studies, where tinnitus cohorts had variable hearing loss profiles, further supports a striatal gating model of tinnitus awareness where the limbic system may be driving tinnitus severity^18^. The striatum is believed to be normally restrictive, blocking out phantom percepts, but becomes dysfunctionally permissive in chronic tinnitus. Initial evidence in support of this model stems from results of acute direct stimulation of the striatum at the junction of the head and body subdivisions (area LC) during deep brain stimulation (DBS) surgery in movement disorders patients with chronic tinnitus, where auditory phantom loudness can be modulated^14^. In those without tinnitus, caudate DBS can trigger auditory phantom percepts^15^ and vascular infarction of the dorsal striatum results in enduring tinnitus loudness reduction^16^. Furthermore, chronic caudate DBS has been shown to significantly improve TFI in some patients with severe, chronic tinnitus^18^. Although the exact physiological mechanisms are not clear, alteration of excitation and inhibition balance either within the caudate nucleus or in its connections to auditory cortex may be modulating phantom percept gating permissiveness^38,39^. Findings from this study extend generalizability of results from human neurophysiological^14,15,18^ and resting-state fMRI^24,25^ studies in binaural patients.

Other tinnitus neuroimaging studies using EEG methodologies have yielded interesting insights that are relevant to our findings. Tinnitus severity response to Tinnitus Retraining Therapy^40^, as measured by the Tinnitus Handicap Inventory^41^ (THI) score, is positively correlated with pre-treatment activities of the left insula^42^. Increased functional connectivity between the caudate and the insula observed in this study suggests that the left insula may be an important structure within corticostriatal loops that link the auditory system with language networks and the limbic system^43,44^. Tinnitus awareness burden is negatively correlated with delta band activity of rostral and dorsal anterior cingulate cortices. Those areas are considered to be at the core of a descending noise cancellation system whose dysfunction may contribute to the percentage of daytime tinnitus awareness^45^. Increased striatal functional connectivity between the left caudate and the right supplementary motor area (SMA) observed in this study may possibly include neighboring dorso-rostral anterior cingulate cortices, thereby contributing to overall tinnitus distress.

The other key finding is significant relationships between right caudate connectivity with non-auditory brain regions and TFI subscale scores. Increased connectivity with the cuneus of the default mode network is correlated with increased difficulty to relax, indicating heightened introspection of the auditory phantom^24^. Increased connectivity with the superior lateral occipital cortex and anterior supramarginal gyrus is correlated with reduced sense of control over the phantom percept, regions that are part of task-positive visual and dorsal attention networks, suggesting enhanced attentional engagement for control of tinnitus^46^. Although the right caudate connectivity shows significant correlation with TFI subscale scores, it did not survive across cohort differences in functional connectivity, most likely due to the underlying small sample size. Nevertheless, the highest connectivity of the right caudate is with the left caudate (Fig. 2A), suggesting comparable functionality across the two caudate nuclei.

Abnormal brain regions identified by resting-state fMRI in this report may serve as biomarkers of tinnitus treatment response or serve as targets for brain-based neuromodulation approaches to mitigate troublesome tinnitus in SSD. For biomarker validation, auditory perceptual training may be adapted for clinical deployment to mitigate tinnitus^47^. Treatment response may be expressed as change in pre-treatment functional connectivity between the striatum and other brain regions, consistent with observations in the insula and anterior cingulate cortices^42,48^. For biomarker targeted brain-based neuromodulation, innovative treatments to disrupt increased corticostriatal functional connectivity^49,50^ may be considered. Techniques include caudate nucleus neuromodulation by direct electrical stimulation^14,15^, MR-guided high intensity focused ultrasound^51^, transcranial stimulation^43,52^ and gamma knife radiosurgery^53,54^.

There are several limitations to this study. Foam earplugs with a noise reduction rating of 32 dB help to mitigate MRI scanner noise, with a spectrum estimated to have a maximum frequency of 1.4 kHz and a peak amplitude of 131 dB over a 10 ms time window^55^. Nonetheless, tinnitus percepts could have been partially masked during data acquisition by noise delivered to the only hearing ear^56,57^. The sample size consists of 15 participants in each cohort that are well matched for gender, age, handedness, degree of hearing loss, and duration of deafness, but with heterogeneity in tinnitus laterality. The relatively small numbers in each cohort and inhomogeneous tinnitus lateralization may be contributory factors to our inability to observe increased right caudate connectivity with auditory cortex and left caudate connectivity with non-auditory brain regions that correlate with TFI subscales. The study design uses a conservative TFI cut-off ≥13 to partition the two cohorts, ensuring NO TIN cohort participants do not have bothersome tinnitus^37^. However, this procedure contributes to TFI score heterogeneity in both cohorts. A future study with larger numbers of participants controlled for tinnitus laterality, and absolute adherence to no tinnitus whatsoever for the NO TIN cohort and the lower bound score of TFI-defined intervals that categorize tinnitus severity^58^ for the TIN cohort would be ideal, subject to participant accrual success.

In conclusion, adults with bothersome tinnitus in acquired SSD exhibited increased functional connectivity between the caudate and auditory cortex, adding evidence to support a striatal gating model of tinnitus, where a dysfunctionally permissive caudate nucleus enables auditory phantoms to reach perceptual awareness. The strength of functional connectivity between the caudate and cuneus was correlated with higher interference with the ability to relax. And the strength of connectivity between the caudate and superior lateral occipital cortex, and the caudate and anterior supramarginal gyrus was correlated with the perception of reduced control over the tinnitus percept. Together, these findings suggest that corticostriatal functional connectivity changes in bothersome tinnitus in SSD may serve as biomarkers to monitor treatment response and suggest candidate targets to develop innovative brain-based neuromodulatory approaches to mitigate tinnitus severity.

## Methods

### Study participants

All 30 study participants had normal hearing in one ear and severe or profound hearing loss in the other. There were 15 TIN and 15 NO TIN participants, where a TFI score ≥13 was the cutoff for bothersome tinnitus or more than “not a problem” in accordance with severity categorization^37,58^. TIN participants had chronic (≥1 year), constant, non-pulsatile tinnitus (Table 1). Participants were recruited from Otolaryngology-Head and Neck Surgery and Audiology clinics affiliated with the University of California, San Francisco (UCSF) and a regional chapter of the Acoustic Neuroma Association. All participants completed the TFI instrument and a demographic questionnaire, and underwent standard clinical audiometry to measure pure tone thresholds. A TFI score of zero indicated no tinnitus whatsoever. All participants with tinnitus localized their auditory phantom to the deaf ear. Audiometric thresholds for low (250 and 500 Hz), middle (1000 and 2000 Hz) and high (4000, 6000 and 8000 Hz) frequency bands were averaged separately to assess hearing loss level for each ear. All participants gave written informed consent. The University of California San Francisco Institutional Review Board approved all study procedures (IRB# 13-10587) and experiments were conducted in accordance with the Declaration of Helsinki.

### Tinnitus severity

The TFI total score and subscales scores were calculated for each participant, and subscale scores were normalized to 100 for subsequent correlation analyses with functional connectivity. The eight TFI subscales address intrusiveness of tinnitus, control over the phantom percept, cognitive interference, sleep disturbance, auditory difficulties, interference with relaxation, quality of life, and emotional distress^37^.

### MRI data acquisition

Imaging data were acquired using a 3-Tesla MRI scanner (Discovery MR750, GE Medical system, Waukesha, WI) to collect both high-resolution structural T1-weighted fast spoiled gradient echo brain volume images (120 axial slices, field of view = 512 × 512 mm, repetition time = 7,232 ms, echo time = 2.78 ms, in-plane voxel dimensions 0.5 × 0.5 mm, slice thickness = 1.5 mm) and to collect spontaneous fMRI data using a resting-state echo planar imaging (EPI) sequence (1.88 × 1.88 mm, 3.0 mm slice thickness, repetition time = 2000 ms, echo time = 28 ms, 100 repetitions). Foam earplugs with a 32 dB noise reduction rating were inserted into both ears during data acquisition.

### Data preprocessing

Resting-state fMRI data were spatially preprocessed using a standardized pipeline implemented in the CONN toolbox. Imaging data preprocessing steps were as follows: functional realignment and unwarping, translation to center, outlier detection using Artifact Detection Tools (https://www.nitrc.org/projects/artifact_detect/), tissue segmentation, spatial normalization to the Montreal Neurological Institute (MNI) template, and spatial smoothing (using a 8 mm FWHM kernel). Prior to functional connectivity analyses, resting-state data were temporally filtered (0.008Hz-0.09 Hz bandpass) and denoised by applying a regression model using 12 realignment parameters and the global mean signal of the white matter.

### Functional connectivity analyses and group statistics

All functional connectivity and group analyses were performed using the CONN toolbox. For resting-state functional connectivity, four seed regions (right/left Heschl’s gyrus (HG); right/left caudate) were anatomically defined using AAL labelled regions (http://neuro.imm.dtu.dk/wiki/Automated_Anatomical_Labeling) as implemented in the CONN toolbox. Correlation coefficients were computed across all voxels of these pre-defined regions of interest (ROIs) with the rest of the brain. Voxelwise regression analyses were performed only within the TIN cohort (n = 15) between functional connectivity and TFI subscale scores. Voxelwise analyses within and between groups (TIN versus NO TIN) were performed using parametric one-tailed t-tests for determining regions with increased and decreased connectivity separately for each of the four seeds. We report on within and across-group whole-brain analyses that survived a threshold of p < 0.05 following a false discovery rate based cluster-mass correction for multiple comparisons^59^.

## Data Availability

The datasets generated during and/or analyzed during the current study are available from the corresponding author on reasonable request, subject to University of California applicable data release policies, rules, and regulations.
