# Author Correction: Corticostriatal functional connectivity of bothersome tinnitus in single-sided deafness

**DOI:** 10.1038/s41598-021-83101-7

**Published:** 2021-02-05

**Authors:** Jennifer Henderson-Sabes, Yingying Shang, Philip L. Perez, Jolie L. Chang, Seth E. Pross, Anne M. Findlay, Danielle Mizuiri, Leighton B. Hinkley, Srikantan S. Nagarajan, Steven W. Cheung

**Affiliations:** 1 University of California, San Francisco, USA; 2 Department of Otorhinolaryngology, Peking Union Medical College Hospital, Beijing, China; 3 Department of Radiology and Biomedical Imaging, University of California, San Francisco, USA

Correction to: *Scientific Reports* 10.1038/s41598-019-56127-1, published online 20 December 2019

The original version of this Article contained an error.

The link provided in the *Functional connectivity analyses and group statistics* section is no longer functional. Therefore, the text,

“For resting-state functional connectivity, four seed regions (right/left Heschl’s gyrus (HG); right/left caudate) were anatomically defined using AAL labelled regions (http://neuro.imm.dtu.dk/wiki/Automated_Anatomical_Labeling) as implemented in the CONN toolbox.”

now reads,

“For resting-state functional connectivity, four seed regions (right/left Heschl’s gyrus (HG); right/left caudate) were anatomically defined using AAL labelled regions^1^ as implemented in the CONN toolbox.”

This has been corrected in the PDF and HTML versions of the Article.
